# Effect of percutaneous assisted approach on functional rehabilitation for total hip replacement compared to anterolateral approach: study protocol for a randomized controlled trial

**DOI:** 10.1186/1745-6215-15-392

**Published:** 2014-10-08

**Authors:** Claudia Hendrickx, Willem De Hertogh, Ulrike Van Daele, Peter Mertens, Gaetane Stassijns

**Affiliations:** Faculty of Medicine and Health Sciences, University of Antwerp, Universiteitsplein 1, 2610 Antwerp, Belgium; Orthopedic Department, ZNA Middelheim, Lindendreef 1, 2020 Antwerp, Belgium; Department of Physical Medicine and Rehabilitation, Antwerp University Hospital, Wilrijkstraat 10, 2650 Edegem/Antwerpen, Belgium

**Keywords:** Total hip replacement, Approach, Gluteus medius, Rehabilitation, Functional outcome

## Abstract

**Background:**

The anterolateral approach is a commonly used technique for total hip replacement. It requires the detachment of a large part of the gluteus medius muscle. However, it is known that this muscle has a great impact on hip stability. Using the percutaneous assisted approach the damage to the gluteus medius can be limited. The purpose of this study is to compare the effect of the percutaneous assisted approach with the anterolateral approach on postoperative functional outcome.

**Methods/Design:**

This study uses a prospective, randomized, parallel-group design with blinded assessment and unblinded treatment to compare the percutaneous assisted approach with the anterolateral approach in total hip replacement surgery. The postoperative results of patients operated on using the percutaneous assisted approach will be compared with those of patients operated on using the anterolateral approach. Prior to surgery patients will undergo baseline measurements. These will consist of gluteus medius measurements (surface-electromyography, strength measurements of abductors and quadriceps and the Trendelenburg test), questionnaires (Oxford Hip Score and 36-item Short Form Health Survey) and functional measures (the Timed Get-Up-and-Go test, Five times Sit-to-Stand test and Six-Minute Walk test). These measurements will be repeated four and 12 weeks after surgery. After surgery both groups will receive usual care.

**Discussion:**

The gluteus medius is the main stabilizer of the hip joint. Therefore, we assume that functional outcome and gluteus medius function of patients after the percutaneous assisted approach will be better than after the anterolateral approach.

**Trial registration:**

This trial was registered with ClinicalTrials.gov on 8 January 2014, registration number: NCT02032017.

## Background

Total hip replacement (THR) is one of the most common orthopedic operations. Based on implant sales figures in the Benelux region of Europe (Belgium, The Netherlands and Luxembourg), the number of THR surgeries performed in 2001 has been estimated at 40,000 (1.52 THR per 1,000 inhabitants per year) [1]. Every THR has an estimated cost of €9000 on average [2] and the length of hospital stay and postoperative care are contributing factors to these costs [2].

Improvements in health-related quality of life (HRQoL) after surgery are reported. However, significant differences between patients and age- and sex-matched controls are noted. An example of this is the scores of the 36-item Short Form Health Survey (SF-36) subscales physical functioning (PF) (*P* = 0.01), role physical (RP) (*P* = 0.05) and vitality (*P* = 0.05) [3]. These lower scores suggest that although patients demonstrate full recovery on psychosocial and pain scales, a deficit in physical functioning remains. This lack of functional recovery has also been found in a review by Vissers *et al*. stating that physical functioning in patients (measured by self-perceived function, clinical measures and actual daily activity) had only recovered to about 80% of that of controls [4]. Furthermore, Rasch *et al*. found persisting muscle atrophy two years after surgery in the acting muscles around the hip [5]. This was caused by loss of muscle volume as well as infiltration of fat tissue.

Postoperative outcome can be influenced by the applied surgical procedure. Two approaches are used to perform THR, with the posterior approach being most commonly used in the USA. Leg lengthening and an increased dislocation rate are the downsides [6–8]. A popular technique in Europe is the anterolateral approach (AA). By using this, a large part of the gluteus medius muscle is released to obtain good access to the acetabulum. There are little or no dislocations reported [9], but limping during the first three months is a well-known problem [6, 7].

**Figure 1:**
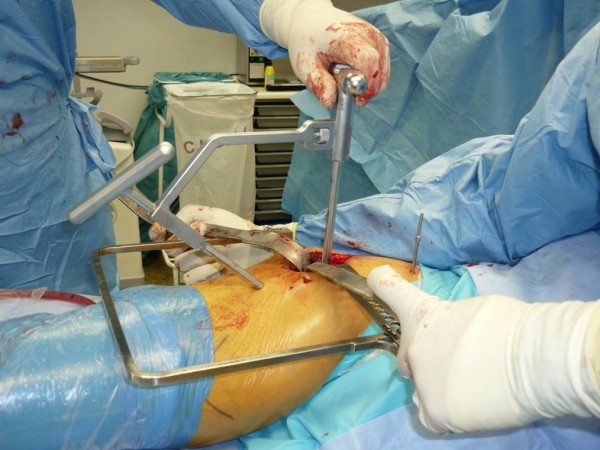
**Percutaneous assisted approach, side view.**

**Figure 2:**
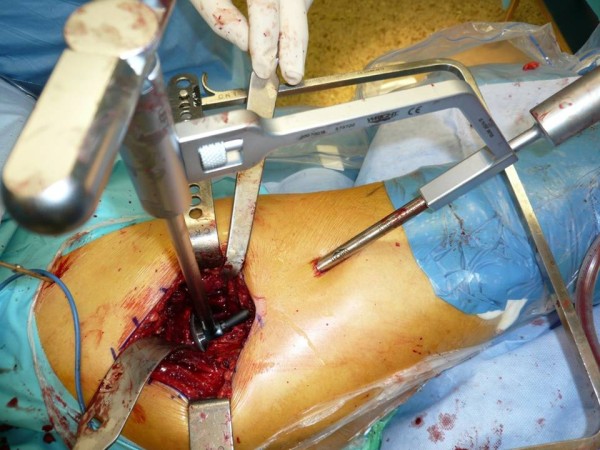
**Percutaneous assisted approach, top view.**

Muscles are major contributors to hip contact force, with gravitational and centrifugal forces combined contributing less than 5%. One of the most important muscles for this is the gluteus medius [10]. It is responsible for stabilizing the femoral neck in the acetabulum during the different stages of gait [11, 12]. This makes it a very important muscle for normal functioning and gait. The gluteus medius is often already weakened before surgery in patients with osteoarthritis of the hip [13]. Since the AA detaches approximately two thirds of the gluteus medius, it affects normal function. Consequently, it can be assumed that interventions sparing the gluteus medius are probably beneficial for patients’ postoperative functional outcome.Damage to the gluteus muscle can be limited using the percutaneous assisted approach (PAA). In this technique, a second small incision (1 cm) at the anterior border of the femur is made. A canula is placed underneath the muscle and used to pass the reamers in the direction of the acetabulum (Figures 1 and 2). There is no need to enlarge the skin incision or to release more muscle insertion to achieve good working access to the acetabulum. There are two advantages of this procedure: sparing of the gluteus medius muscle and safe access to the acetabulum to obtain perfect positioning of the implants.

Interventions sparing the gluteus medius are probably beneficial for patients’ postoperative functional outcome. The purpose of this study is to evaluate the effect of the PAA on postoperative functional outcome compared to the AA.

## Methods/Design

### Study design

This study uses a prospective, randomized, parallel-group design with blinded assessment and unblinded treatment to compare the PAA with the AA in THR surgery.

### Ethical approval

Full ethical approval has been given by the ethics committee of the Antwerp University Hospital (UZA) and Hospital Network Antwerp (ZNA) (approval number: B300201318915).

### Patient selection

Patients will be recruited at the orthopedics department of the ZNA Middelheim hospital in Antwerp, Belgium. Patients with a hip complaint for which THR is indicated are screened for eligibility by a specialized surgeon (PM). Eligible patients will be between 50 and 80-years-old and suffer from unilateral hip arthritis or avascular necrosis in need of THR. Comorbidities affecting functional outcome will be used as exclusion criteria. These consist of symptomatic lumbar pathology or those in need of surgery or intervention on the ipsilateral knee and/or ankle or foot. Additionally, neurological disorders, according to the International Classification of Diseases, such as Parkinson’s disease and previous cardiovascular accidents (CVA) are excluded from this study.

Eligible patients who provide informed consent will be sent to the Antwerp University Hospital (UZA) where an appointment for preoperative (baseline) measurements will be made. These consist of gluteus medius measures, HRQoL-questionnaires and functional measures. Patients will be given an information sheet and a written informed consent will be obtained prior to baseline measurements.

### Gluteus medius measures

#### Surface-electromyography of gluteus medius muscle

Patients will perform a single leg stance (SLS) and a maximal voluntary isometric contraction (MVIC) of the gluteus medius during surface-electromyography (SEMG) measurement. Both have shown excellent reliability (SLS intraclass correlation coefficient (ICC) = 0.93; MVIC ICC = 0.98) [14]. Electrodes will be positioned 2.5 cm posterior of the middle of the line between the top of the iliac crest and the greater trochanter. This is more posterior than the method of Hermens *et al*. [15]. This decision is based on a cadaver-study by Van Leemput *et al*. (Van Leemput W, Collier E, Saeys N, Stassijns G: **Measuring tensor fasciae latae and gluteus medius with surface EMG: a cadaver-based study.** Unpublished). They demonstrated that the distance from the needle tract to the central point of the muscle was significantly less when the electrodes were placed more posterior (*P* <0.0001). Therefore we prefer this method.

#### Quadriceps and abductor muscle strength

Quadriceps and abductor strength will be measured with the microFET 2 hand-held dynamometer (Biometrics Motion nv, Almere, The Netherlands). Measurements with this device are highly reliable, with excellent intrarater reliability (ICC 0.921 to 0.929) and valid, with high correlations with the Timed Get-Up-and-Go test (TGUG) (r = -0.710 to -0.862) and gait speed (r = 0.728 to 0.825) [16].

For quadriceps force the patient is seated with the knees bent 90°. Resistance will be administered on the ventral side of the lower leg, just proximal of the ankle joint. Abductor force will be measured in a supine position. Resistance will be administered on the lateral side of the upper leg, just proximal of the knee joint. Patients will be asked for a MVIC. The tests will be repeated three times. The mean value of the three measurements will be recorded.

#### Trendelenburg test

This test measures abductor force in a functional way. The patient is asked to raise one leg (sound side) and raise the non-stance side of the pelvis as high as possible for 30 seconds. The response is classified as followed [17]:

1. Normal: The pelvis on the non-stance side can be lifted maximally for 30 seconds.
2. The pelvis on the non-stance side can be lifted but not maximally
3. The pelvis is elevated but not maintained for 30 seconds
4. No elevating of the pelvis on the non-stance side
5. Drooping of the pelvis
6. Non-valid response: due to hip pain or uncooperative patient

Test-retest reliability coefficients (κ) are found to be greater than 0.75 [18].

### HRQoL-questionnaires

All patients will be required to complete the Oxford Hip Score (OHS) and the 36-item Short Form Health Survey (SF-36) questionnaires. The OHS is a disease-specific questionnaire that consists of 12 questions for the evaluation of pain and hip function in relation to various activities. Each question contains five quantifiable answers, leading to a total score that can range from 12 (least problems) to 60 (most problems). For our study population we will use the Dutch version. This version is believed to have excellent test-retest reliability (ICC = 0.97) and a very high correlation with the visual analogue scale score of the hip (r ≥ 0.7) [19]. Changes in the OHS are also closely related to the patients’ satisfaction with their surgery [20, 21]. Furthermore, it is very sensitive to change in the first postoperative year [19].

The SF-36 is a generic questionnaire that contains 36 items measuring health on eight different dimensions. These dimensions cover functional status, wellbeing and overall evaluation of health. It draws attention to broader problems of physical functioning than the OHS [22]. The SF-36 is not very sensitive to individual changes, but is effective for measuring group changes, so it becomes a good instrument for scientific research [23]. Internal-consistency reliability of the Dutch version meets the 0.70 level (Cronbach’s alpha) recommended for group comparisons on all scales (range: 0.78 to 0.92). The PF and Bodily Pain (BP) scales even met or exceeded the 0.90 level recommended for individual comparisons [24].

### Functional measures

All patients will have functional measurements recorded using the TGUG test, the Five Times Sit-to-Stand test (5tSTS) and the Six-Minute Walk (6MWT) test. In the TGUG test, the participant is asked to stand up and walk 3 m, turn around and walk back to the chair and to sit down. The score recorded is the time used to complete the test expressed in seconds [25]. Yeung *et al*. investigated the psychometric properties of the TGUG test in inpatients on an orthopedic rehabilitation ward [26]. They found a high ICC (0.80). The standard error of measurement was 10.2 seconds. Change in TGUG scores correlated with the changes in pain (r = 0.21; *P* <0.01) and function (r = -0.23; *P* <0.01).

The 5tSTS test is an easily feasible test where the patient has to stand up and sit back down five times as quickly as possible is a good predictor of falling. A worse score (a longer time needed to complete the test) on the 5tSTS implies a greater chance of falling [27]. Furthermore it predicts problems with daily activities [28]. The test-retest reliability is good to high (ICC 0.64 to .096) in most populations and settings and validity is scored good (compared to 1 Repetition Maximum (1RM) leg press, Pearson correlation coefficient r = 0.68, *P* <0.05) [29, 30]. The standard error of measurement (6.3% of mean 5tSTS) and the minimum detectable change (17.5% of mean 5tSTS) are low [31].

The 6MWT requires a 30 m hallway, but no exercise equipment or advanced training for technicians. The test measures the distance a patient can quickly walk on a flat, hard surface in a time-period of six minutes (the six-minute walking distance (6MWD)). The test will be carried out according to the American Thoracic Society guidelines [32]. This means there will be a standard instruction and demonstration prior to the test; the researcher is not allowed to walk with the patient. No verbal feedback is given. Every minute the remaining time is mentioned with a standard phrase of encouragement, spoken in a neutral tone. The timer is started when the patient starts walking. The test-retest reliability is excellent (ICC 0.95). The 6MWD is significantly greater for active than for inactive adults (*P* <0.0001). It moderately correlates with chair stands (r = 0.67), standing balance (r = 0.52), gait speed (r = -0.73) and self-reported physical functioning (r = 0.55) [33].

### Intervention

All patients will get a standard THR (cementless hydroxyapatite-coated cup and a titanium plasma-sprayed stem (Wright Medical, Warshaw, USA)) with a ceramic-on-ceramic (third generation BIOLOX™ delta (Wright Medical, Warshaw, USA) couple. Preoperative leg length and offset are marked to reconstruct the preoperative leg length and to obtain the optimal offset.

In the AA group, a standard transgluteal approach is used and the most optimal component position is achieved. In the PAA group, a muscle sparing technique is used. This will not compromise the component position, but fewer release and detachment of the gluteus medius is necessary. Both groups will receive usual care (UC) after surgery. This includes standard physiotherapy care consisting of mobilizing and strengthening techniques. All patients will receive a booklet containing information about the surgery, weight-bearing capacity after the surgery and rehabilitation in general.

### Follow-up measurements

All measurements will be repeated at four weeks and three months post-surgery. Six weeks post-surgery patients will be seen by the orthopedic surgeon for clinical and radiographic control. These are routine measurements to assess the postoperative course.

### Randomization, blinding and treatment allocation

Patients will be allocated to one of two treatment groups by the surgeon at the preoperative consultation. Randomization will take place by means of blinded envelopes. In total 10 envelopes are provided: five that allocate the patient to the AA group and five that allocate the patient to the PAA group. This is to ensure that the same number of patients is randomized in one of the two treatment groups. Patients receive the same information from the surgeon regardless of their group allocation. A blinded researcher will perform baseline and follow-up measurements. To ensure blinded assessment, SEMG will be carried out by an independent researcher. This because blinding is not possible due to different locations of scar tissue.

### Power and sample size calculation

Starting with a pilot study, we will include five people in each group. On this basis we will do power and sample size calculations in order to commence with the randomized controlled trial.

### Analysis

The primary outcome measure will be the score on the TGUG. Secondary outcome measures will include SEMG, strength of quadriceps and gluteus medius, OHS and SF-36 questionnaire score, score on the 5tSTS, the Trendelenburg test score and the 6MWT score. Normality of data will be checked via the Kolmogorov-Smirnov test. Differences between both treatment groups at baseline and follow-up measurements will be analyzed using the chi-square statistical method for categorical variables. For continuous variables, comparisons between both groups’ baselines will be made using an independent samples t-test. Comparisons between both groups at the follow-up measurements will be made using a two-factor repeated measures Analysis of Variance (ANOVA) (group × time). Baseline values will be included as the first follow-up measurements. Differences between successive measurements within a treatment group will be analyzed using a one-factor repeated measures ANOVA. Significance value for all tests is set at *P* <0.05. The results of all subjects will be analyzed, regardless of their treatment adherence (intention-to-treat analysis).

## Discussion

The aim of this study is to assess the difference in functional outcome after THR through the PAA, compared to THR through the AA. The main difference between these two approaches is their impact on the gluteus medius. Whereas the AA detaches two thirds of the gluteus medius, the PAA only detaches a small part of the muscle. Since the gluteus medius is one of the most important muscles for the stability of the hip joint [10–12], we hypothesize that functional outcome is better or is achieved faster if THR surgery is performed through the PAA instead of the AA.

For this study we chose to have multiple outcome measures (SEMG, strength of gluteus medius and quadriceps, the Trendelenburg test score, the 6MWD and scores on the OHS, SF-36, TGUG and 5tSTS tests) to be certain we measure all possible effects. According to Vissers *et al*. three aspects should measure functional outcome: self-perceived function, functional tests in a laboratory setting and actual daily activity [4]. Both self-perceived function (measured by the OHS and SF-36 questionnaires) and functional tests (measured by the 6MWT and the TGUG, 5tSTS and tests) are use as outcome measures in our study. However, we chose not to assess actual daily activity at this stage. Rasch *et al*. reported remaining muscle atrophy up to two years after THR [5]. Therefore, we chose to include muscle strength measurements (measured by the SEMG, MicroFET and Trendelenburg tests). If this protocol takes too much effort for our patients it is possible that we could reduce it by removing the 6MWT and the Trendelenburg test.

This study has the following limitations. First, complete blinding cannot be achieved because of the SEMG measurements of the gluteus medius and the difference in location of scar tissue for both surgical approaches. Therefore, we chose to have an independent researcher carry out SEMG measurements and note the results, ensuring the other measures will be blinded. Second, we will only evaluate the short-term outcome. Because of the difference in approach we assume that the intervention group will already show a great deal of improvement in the short-term, while the control group will improve less. In later studies it will be necessary to investigate long-term success as well.

The following strengths can be mentioned. First, we conduct a single centre study, ensuring a standardized approach. Second, we use a wide range of outcome measures allowing us to achieve a total picture of the rehabilitation process. This is why patients are asked to complete both a generic (SF-36) and a disease-specific questionnaire (OHS). The disease-specific questionnaire is believed to better measure changes in function. The generic questionnaire is used to detect changes in psychosocial terms as well.

## Trial status

Statistical analysis. Recruitement began October 2013 and was finished in September 2014.

## Abbreviations

1RM: One repetition maximum
5tSTS: Five times Sit-to-Stand
6MWD: Six-minute walking distance
6MWT: Six-Minute Walk test
AA: Anterolateral approach
ANOVA: Analysis of Variance
BP: Bodily Pain
CVA: Cerebrovascular accident
HRQol: Health-related quality of life
MVIC: Maximal voluntary isometric contraction
OHS: Oxford Hip Score
PAA: Percutaneous assisted approach
PF: Physical functioning
RP: Role physical
SEMG: Surface-electromyography
SF-36: 36-item Short Form Health Survey
SLS: Single leg stance
TGUG: Timed Get-Up-and-Go
THR: Total hip replacement
UC: Usual care
UZA: Universitair Ziekenhuis Antwerpen (Antwerp University Hospital Antwerp)
ZNA: Ziekenhuis Netwerk Antwerpen (Hospital Network Antwerp).
